# Biomarkers in renal cell carcinoma and their targeted therapies: a review

**DOI:** 10.37349/etat.2023.00175

**Published:** 2023-10-25

**Authors:** Shruti Gupta, Shamsher Singh Kanwar

**Affiliations:** University of Toronto, Canada; Department of Biotechnology, Himachal Pradesh University, Summer Hill, Shimla 171 005, India

**Keywords:** Renal cell carcinoma, biomarkers targeted therapies, molecular medicine, DNA methylation, microRNA, long noncoding RNA

## Abstract

Renal cell carcinoma (RCC) is one of the most life-threatening urinary malignancies displaying poor response to radiotherapy and chemotherapy. Although in the recent past there have been tremendous advancements in using targeted therapies for RCC, despite that it remains the most lethal urogenital cancer with a 5-year survival rate of roughly 76%. Timely diagnosis is still the key to prevent the progression of RCC into metastatic stages as well as to treat it. But due to the lack of definitive and specific diagnostic biomarkers for RCC and its asymptomatic nature in its early stages, it becomes very difficult to diagnose it. Reliable and distinct molecular markers can not only refine the diagnosis but also classifies the tumors into thier sub-types which can escort subsequent management and possible treatment for patients. Potential biomarkers can permit a greater degree of stratification of patients affected by RCC and help tailor novel targeted therapies. The review summarizes the most promising epigenetic [DNA methylation, microRNA (miRNA; miR), and long noncoding RNA (lncRNA)] and protein biomarkers that have been known to be specifically involved in diagnosis, cancer progression, and metastasis of RCC, thereby highlighting their utilization as non-invasive molecular markers in RCC. Also, the rationale and development of novel molecular targeted drugs and immunotherapy drugs [such as tyrosine kinase inhibitors and immune checkpoint inhibitors (ICIs)] as potential RCC therapeutics along with the proposed implication of these biomarkers in predicting response to targeted therapies will be discussed.

## Introduction

As per International Agency for Research and Cancer, a 22% increase has been observed in the number of people diagnosed with kidney cancer [1]. Kidney cancer is now amongst the top ten most common cancers in males and has taken the fourteenth place worldwide based upon its incidence in both genders. Amongst all the kidney cancers, renal cell carcinomas (RCCs) which originates from renal epithelium accounts for 90% of all cases and are the most lethal [2]. RCC is a highly vascularized cancer where approximately 30% of patients display metastasis when diagnosed and a similar percent of patients display reoccurrence post-surgery though they were diagnosed initially with a clinically localized disease [3]. According to Dabestani et al. [4], the reoccurrence time ranged from 12.5 months to 43.6 months on the basis of different risk categories [4]. RCC has been categorized into seven different subtypes. Amongst these, clear cell RCC (ccRCC) accounts for the most common subtype. The other two common subtypes include papillary RCC (pRCC) and chromophobe RCC (chRCC) [5, 6]. Modern topographic and ultrasound techniques are capable of diagnosing renal tumors in the early stages. However, due to lack of early symptoms of the disease, the diagnosis of RCC usually occurs incidentally through radiology imaging techniques while identifying other medical conditions [7]. Verification of the pathological phase and type of the cancer, as well as its timely diagnosis is very important for effective management of the disease. Biomarkers have imparted an advancement in the understanding of the scope of different malignancies with applications in screening, diagnosis, and prognosis of the disease as well as their experimental and analytical epidemiology [8]. In the past years, researchers have discovered the potential role of bio-markers in RCC. Several biomarkers have been proposed to predict the risk of RCC recurrence. Biomarkers can incredibly change the way RCC is diagnosed and provide a cost-effective screening of high-risk patients. They also display potential roles in the identification of aggressive cancers as well as the determination of the possibility of recurrence post-surgery with minimal imaging and thus providing targeted therapies for patients with metastatic RCC [9]. In the present study, we attempt to discuss the current situation of the use of biomarkers in diagnosing and prognosis of RCC, as well as the proposed clinical implications of these biomarkers in targeted therapies.

## Molecular understanding of RCC

RCC involves a broad spectrum of molecularly and morphologically distinct cancer subtypes, all of which originate from the kidney epithelium [10]. It has been characterized by poor diagnosis due to lack of early warning symptoms, resistance to chemo and radiation therapy, diverse clinical expressions as well as exceptional responses to interferon-α (IFN-α) and interleukin-2 (IL-2) like immunotherapeutic agents [11]. Several laboratories and consortiums including The Cancer Genomics Atlas (TGCA) have provided an extraordinary understanding of the molecular basis of RCC pathobiology through several studies [12]. Prior investigations suggested that frequent mutations and inactivation of the von Hoppel Lindau (*VHL*) gene, which is responsible for vascular endothelial growth factor (VEGF) and mammalian target of rapamycin (mTOR) inhibitor, is a major factor in RCC, specifically ccRCC. Mutations in VHL results in hypoxia inducing factor (HIF) protein accumulation that up-regulates the VEGF pathway which plays a role in angiogenesis, tumor cell migration, proliferation, and permeability. Besides VEGF, delta like canonical notch ligand 4 (*DLL4*) is also considered as a prognostic gene in RCC [13]. Other mutations that have been identified as responsible for RCC through genome sequencing studies include *BRCA1* associated protein 1 (*BAP-1*) which helps control cell division, cell growth, and cell death; polybromo-1 (*PBRM1*) that codes for an ATP dependent chromatin remodelling protein; set domain-containing protein-2 (*SETD2*) responsible for the production of histone methyltransferase; and phosphatidylinositol-4,5 bisphosphate 3-kinase gene (*PIK3CA*) that imparts directions for producing p110 protein, a subunit of the phosphatidylinositol-3 kinase enzyme [14]. Molecular studies of ccRCC have demonstrated that large deletion of chromosome 3p which contains the second copy of the *VHL* gene also results in deletion of tumor suppressor genes *PBRM1*, *SETD2*, and *BAP-1*. Additional chromosomal aberrations, including gain of 5q, loss of 9p as well as 14q are often linked with tumor cell progression [15]. A renal cancer associated gene, renal cancer differentiation gene 1 [*RCDG1*, originally called as chromosome 4 open reading frame 46 (C4orf46)] is significantly down regulated in RCC tissues as compared to normal adjacent tissues [3].

In the Heidelberg classification of RCC, pRCC was identified as a distinct entity. Several genetic studies demonstrated aberrations in the mesenchymal-epithelial transition factor (*MET*) gene to be the major reason for maximum cases of pRCC [16]. Mutations in *MET* are responsible for 13–15% of non-heritable pRCC. The germline mutation in the gene encoding fumerate hydratase (*FH*; a protein of the tricarboxylic acid cycle) is responsible for hereditary leiomyomytosis and RCC. Mutations in the genes such as cullin-RING E3 ubiquitin ligases (*CUL3*) and nuclear factor erythroid 2-related factor 2 (*NRF2*) that regulate *NRF2*/antioxidant response element (*NRF2-ARE*) have been observed in the sporadic pRCC [17]. Recently, 12 recurrently mutated genes including telomerase reverse transcriptase (*TERT*), AT-rich interaction domain 1A (*ARID1A*), lysine demethylase 6A (*KDM6A*), lysine methyltransferase (*KMT2D*), *NRF2* (also called *NFE2L2*), *MET*, adenomatous polyposis coli (*APC*) and codes for tumor protein p53 (*TP53*) were identified to be responsible for both type 1 and type 2 pRCC in a subset of 22 cases by Murugan et al. [18]. Besides *MET* aberrations, most low grade pRCC and a few percentages of high-grade pRCC commonly includes gain of entire chromosome 7 and 17, possible gains of chromosome 112, 16, and 20 as well as loss of Y chromosome [15, 19].

chRCC accounts for just 5–7% of RCC. As compared to other renal cancers, chRCC is associated with complete loss of 7 different chromosomes (i.e. 1, 2, 6, 10, 13, 17, and 21). The cancer genome atlas (TCGA) analysis have conformed characteristic patterns of loss from chromosomes 1, 2, 6, 10, 13, and 17 in 86% of tumors with further loss of chromosomes 3, 5, 8, 9, 11, 18, and 21q in 12% to 58% of tumors. Other than the chromosomal loss, several cohort investigations also revealed multiple chromosomal gains in chRCC. The frequently observed chromosomal gains include that of chromosome 4, 7, 15, 19, and 20 [20]. Molecular studies indicated that p53 mutations account for 20–32% of chRCC cases, phosphatase and tension homolog (*PTEN*) mutations were observed in almost 6–9% of patients, *TERT* promoter mutations/rearrangements in 12% of cases, and mitochondrial DNA alterations were rarely observed [21]. However, studies evaluating the metastasis of chRCC revealed that TP53 mutations, DNA hypermethylation, imbalanced chromosomal duplication, *PTEN* mutations, cyclin dependent kinase inhibitor 2A (*CDKN2A*) mutations have been coupled with high-risk features and poor survival [22]. A recent study conducted by Rogala et al. [23] which included 5 males and 5 females observed mutations of 13 genes viz. codes for the riboendonuclease dicer (*DICER1*), fibroblast growth factor receptor 3 (*FGFR3*), Janus kinase 3 (*JAK3*), suppressor of fused homology (*SUFO*), family with sequence similarity 46, member c (*FAM46C*), Fanconi anemia complementation group G (*FANCG*), phospholipase C gamma 2 (*PLCG2*), DNA polymerase epsilon catalytic subunit A (*POLE*), epithelial cell adhesion molecule (*EPCAM*), mutY DNA glycosylase (*MUTYH*), androgen receptor (*AR*), *APC* and *MET* to be responsible for small cell variant of chRCC.

## RCC biomarkers

Cancer biomarkers form the measurable molecular tools that have the potential for determining the incidence of cancer, risk of cancer, cancer prognosis, patient follow-up as well as predicting the response to therapy. These biomarkers include biomolecules such as DNA, RNA, proteins, or any other biomolecules that can be diagnosed in specimens obtained through biopsies or those obtained through non-invasive approaches such as from blood, urine, buccal swabs, saliva, and stool [24]. In the wake of the improved techniques of high-performance genomics, proteomics, and metabolomics, there has been rapid growth in the investigations studying biomarkers for RCC in recent years. Existing biomarkers for RCC have been classified as tissue-based, urine-based, or blood-based biomarkers on account of their origin [25].

**Figure 1:**
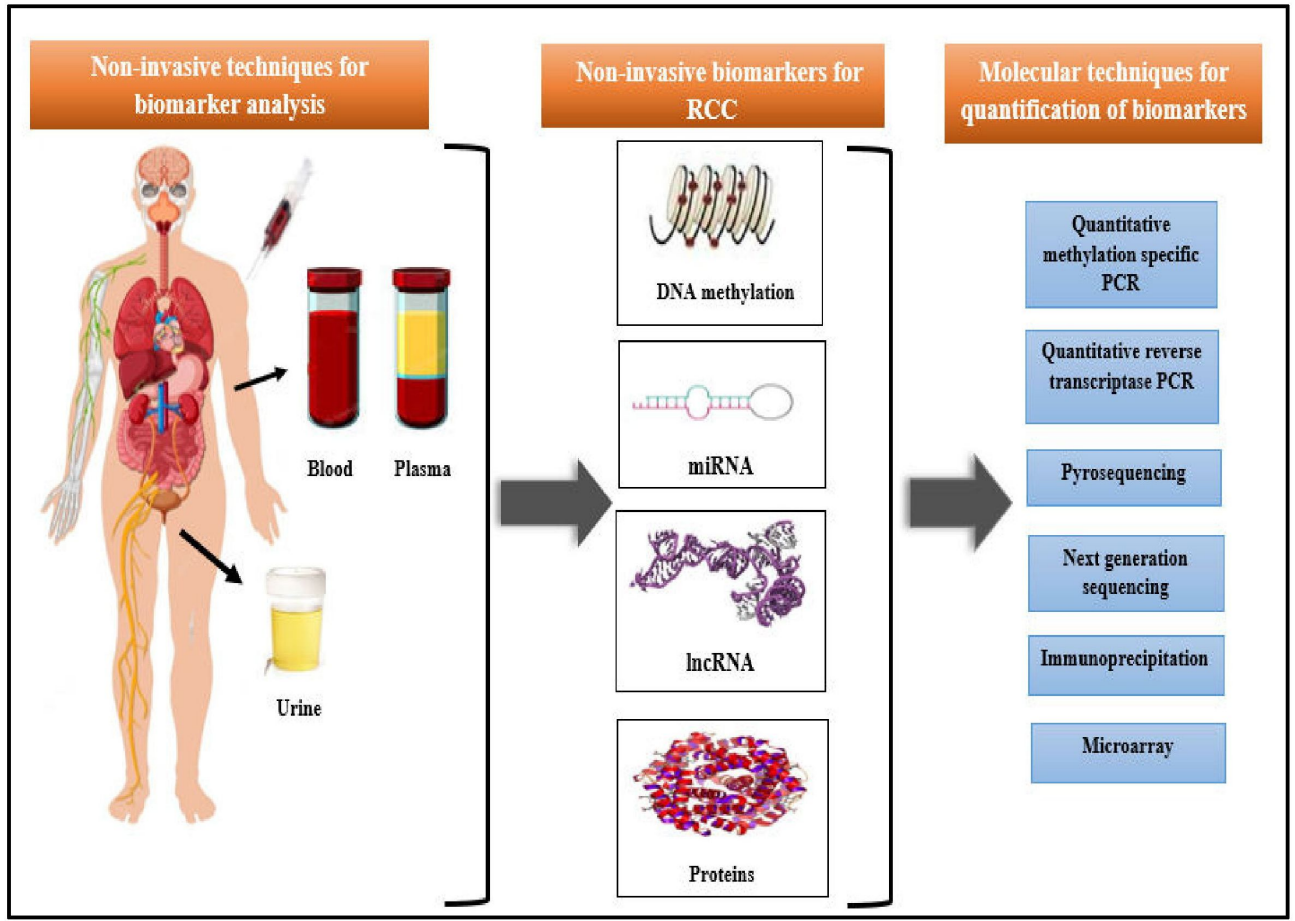
Non-invasive biomarkers for RCC and their quantification techniques

Previous studies have revealed that a prolonged duration (up to 50 years) is essential from initial genetic alterations to the scientific exemplification of RCC tumors (specifically ccRCC) as its clonal expansion is very sluggish. Although there is hardly any evidence of some histological change in the corresponding cytologically normal renal tissue of the patient with renal tumors, the accumulation of epigenetic variations has been observed in such non-cancerous renal tissues (NRTs) thereby recommending them for early diagnosis of RCC. DNA methylation, microRNA (miRNA; miR), and long noncoding RNA (lncRNA) provide as non-invasive epigenetic blood circulating and urine-based biomarkers for the diagnosis of kidney cancer [26]. These epigenetic non-invasive biomarkers can be smoothly perceived in body fluids like peripheral blood and urine samples or through quantitative or qualitative polymerase chain reaction (PCR) techniques (Figure 1) [2].

## DNA methylation as potential non-invasive biomarkers for RCC

Epigenetic alterations such as promoter methylation have played important roles in tumorigenesis by silencing tumor suppressor genes. DNA methylation changes the chemical properties of DNA without altering the chemical sequence. It involves addition of methyl group to the cytosine group of CpG island in the promoter region of genes. Methylation in the promoter region of the gene makes it inaccessible for transcription thereby silencing the genes. DNA methylation has been coupled with clinicopathological features and patient survival. Besides DNA methylation, gene-specific hypomethylation (a decrease in the genome-wide methylation) is another common aberration that may bring about the activation of proto-oncogenes, DNA damage, reactivation of transposons, and genomic instability [24, 27]. An investigation identified promoter methylation of basonuclin 1 (*BNC1*), signal peptide, CUB domain and EGF like domain containing 3 (*SCUBE3*), GATA binding protein 5 (*GATA5*), secreted frizzled-related protein 1 (*SFRP1*), gremilin 1 (GREM1), Ras association domain family 1 isoform A (*RASSF1A*), protocadherin 17 (PCDH17), laudinin 1 (*LAD1*) and neurofilament heavy polypeptide (*NEFH*) coding genes as potential diagnostic and prognostic methylation biomarkers for RCC [28]. In comparison to genetic alterations, DNA hypermethylation is more frequently observed in most RCC subtypes. Another systematic review on DNA methylation biomarkers for RCC studied 15 biomarkers in two autonomous study populations [6]. They studied 15 biomarkers in two autonomous study populations. Similar sensitivities and specificities of DNA hypermethylation biomarkers i.e. *APC*, *CDKN2A* (p16), O^6^-methyl-guanine-DNA-methyltransferase (*MGMT*), retinoic acid receptor β (*RARB2*), *TIMP3 (*tissue inhibitor of metalloproteinase-3), *RASSF1A* and *VHL* were observed in RCC samples in all studies. The biomarkers were studied by methylation specific-PCR in most of these studies. Higher *VHL* methylation was found in patients with ccRCC subtypes as compared to other subtypes [29]. A significantly higher DNA methylation for genes in ccRCC tissues as compared to NRTs was studied by Kubiliutė et al. [30]. The diagnostic procedures illustrated a panel of Zinc finger protein 677 (*ZNF677*), fibrillin 2 (*FBN2*), protocadherin 8 (*PCDH8*), transcription factor AP-2 β (*TFAP2B*), and tachykinin precursor 1 (*TAC1*) biomarkers with 82% sensitivity and 96% specificity. In the tissue samples, detrimental clinicopathologic specifications are notably linked to hypermethylation of *ZNF677* and *PCDH8*. In another comparative study, Kubiliutė et al. [31] performed DNA methylation analysis of potential biomarkers in urine samples from RCC patients and asymptomatic controls through two-colour Human DNA Methylation 1  ×  244K Microarrays and methylation sensitive PCR. The comparison of RCC (specifically ccRCC) and NRT samples revealed significantly higher methylation at the regulatory regions of all investigated biomarkers in ccRCC tissues as compared to NRT tissues.

DNA methylation modifications have also been linked to different tumor stages and clinical prognosis in RCC, however, none of these markers have entered into clinical routine. For example, the methylation of *PCDH8* was coupled to the advanced tumor stage and was strongly predicted for overall survival (OS). *PCDH8* methylation conjointly with *ZNF677* biomarkers displayed a considerably stronger prognostic power [30]. The association of promoter methylation of *SFRP1*, *GATA5*, *NEFH*, *GREM1*, and *BCN1* with survival in RCC was identified by Peters et al. [27]. A prognostic model for ccRCC further adds five DNA methylation markers (*GREM1*, *GATA5*, *LAD1*, *NEFH*, and *NEURL* methylation) to the currently known clinicopathological factors [32]. Recently it was demonstrated that *ZNF582* was noticeably hypermethylated and under-expressed in ccRCC patients Ding et al. [33]. *ZNF582* hypermethylation was pronouncedly associated with prognosis and clinical stage. Experimental data claimed that under-expression of *ZNF582* markedly hindered apoptosis and promoted cell proliferation, migration, invasion, and adhesion of ccRCC [33]. It was further illustrated by Yang et al. [34] that ZNF582 binds to tight junction protein 2 (TJP2) and up-regulates TJP2 protein expression. Elevated TJP2 protein combines with extracellular signal-regulated kinase 2 (ERK2) to promote ERK2 protein expression which suppresses the phosphorylation of ERK2, thereby inhibiting the growth and metastasis of ccRCC [34].

Hypermethylation of CpG islands in promoter regions of genes and overexpression of anti-oxidant pathway within tumor cells have been characterized as markers of poor prognosis of pRCC [16]. Hypermethylation of *RASSF1A* is frequently observed however, hypermethylation of glutathione S-transferase pi 1 (*GSTP1*), cadherin 1 (*CDH1*), and *APC* are infrequent. Also, *CDH1* hypermethylation is also associated with patient survival and the pathological stage of the disease [35]. Progressive methylation changes in several CpGs from localized to advanced stage type II pRCC have been identified by Yang et al. [36]. Four CpG methylation markers (cg00489401, cg27649239, cg20555674, and cg07196505) were identified specifically that differentiated between localized and advanced stage of type II pRCC. Patients’ survival in pRCC was remarkably coupled with several genes including chromosome 19 open reading frame 33 (*C19orf33*), gamma-glutamyltransferase 6 (*GGT6*), GIPC PDZ domain containing family member 1 (*GIPC2*), HERV-H LTR-associating 1 (*HHLA2*), homeobox D3 (*HOXD3*), hydroxysteroid 17-beta dehydrogenase 14 (*HSD17B14*), phospholipase A and acyltransferase 3 (*PLAAT3*), and transmembrane protein 71 (*TMEM71*) that were observed through combined gene expression survival analysis and DNA methylation studies [37] (Table 1).

**Table 1:** DNA methylation biomarkers in RCC and their underlying mechanism

**Biomarker**	**Sample**	**Method of diagnosis**	**Mechanism**	**Reference**
*VHL*	Blood	Restriction endonuclease qPCR	VHL promoter methylation inactivates the VHL tumor suppressor gene which in turn regulates HIF protein and hence contributes to RCC carcinogenesis	[29, 38]
*RASSF1A*	Blood	Restriction endonuclease qPCR; MSP	Hypermethylation of the RASSF1A promoter inactivates the RASSF1A tumor suppressor gene involved in DNA repair, cell cycle, and cell death	[39]
*PCDH17*	Urine, serum, and tissue samples	Quantitative MSP	PCDH methylation was linked to the downregulation of the PCDH17 tumor suppressor gene that functions through the regulation of cell-to-cell adhesion, growth control, and signal transduction PCDH17 hypermethylation was linked to progression and shorter disease-free survival in RCC patients	[39, 40]
*NEFH*	Tissue	RNA expression microarray	DNA methylation of NEFH promoter and loss of expression has been linked to the AKT/β-catenin pathway leading to increased glycolysis rates and changes in the mitochondria	[41]
*APC*	Urine and blood	Quantitative MSP	APC promoter methylation and subsequent loss of expression of the APC gene have been associated with nuclear β-catenin accumulation and p53 deficiency	[42]
*CDKN2A* (p16)	Urine, blood, and tissue	Quantitative MSP	CDKN2A methylation plays an important role in RCC metastasis by affecting the p16/p14 expression	[43]
*MGMT*	Blood, urine, and tissue	Quantitative MSP	Promoter methylation of MGMT inhibits the MGMT DNA repair gene	[44]
*TIMP3*	Blood, urine, and tissue	Quantitative MSP; restriction endonuclease qPCR	Methylation-associated silencing of TIMP3 has been associated with the acquisition of tumorigenesis as TIMP3 contributes to VEGF-mediated angiogenesis regulation	[45]
*ZNF677*	Blood, urine, and tissue	Methylated RNA immunoprecipitation-sequencing (MeRIP-seq) and MeRIP-qPCR	Promoter methylation of ZNF677 leads to ZNF677 silencing which functions as a tumor suppressor	[46]

qPCR: quantitative PCR; MSP: methylation specific PCR; AKT: v-akt murine thymoma viral oncogene homolog

Other than PCR, methylation sensitive restriction enzymes, methylation specific droplet digital PCR, microarray, next genome sequencing, methylation sensitive high-resolution melting, pyrosequencing and methylated DNA immunoprecipitation are the methods in use for identifying epigenetic variants [24]. Although several methylation genes have been analyzed as potential markers for RCC through genome-wide methylation studies, still proper bioinformatic analysis, standardization of methods, and validation on large sets of patients are required to speed up the use of these markers in the diagnosis and treatment of kidney cancers.

## miRNA non-invasive RCC biomarkers

miRNA are small noncoding RNA that form important biomarkers for RCC diagnosis, prognosis, and monitoring. miRNA regulates post-transcriptional gene expression and play roles in cellular functions like apoptosis and proliferation [47]. Changes in their regulatory functions and expressions are the fundamental aspects of various pathogenesis. miRNA have been categorized as oncogenic or tumor suppressive/onco-suppressive based on their tumor-stimulating or inhibiting effect, respectively. The onco-suppressive miRNA targets the mRNA of oncogenes or genes encoding proteins which mediate the progression of kidney tumors, while the mRNA of tumor suppressor genes are the targets of oncogenic miRNA [48]. To illustrate, mesenchymal-epithelial transition factor (*c-MET*) and neurogenic locus notch homolog protein 1 (*NOTCH1*) oncogenes are targets of miR-34a [48].

As revealed by investigations from tumor patients, miRNA are released into the biological fluids such as whole blood, serum, plasma, and urine by malignant cells making them potential non-invasive diagnostic, prognostic as well as predictive biomarkers [49]. They are present stably in different forms such as bound to protein complexes, freely circulating, or in extracellular vesicles. Alterations in the levels of miRNA in biological fluids are coupled with molecular changes that take place in oncological tumors [50]. Expression studies demonstrated that serum expression levels of miR-122-5p and miR-206 were remarkably declined in ccRCC patients as compared to the healthy controls [51]. They proclaimed that high serum levels of miR-122-5p and miR-206 are linked with a brief span of progression-free, cancer specific, and OS in ccRCC patients. Besides, the expression of miR-15a, a tumor suppressor RNA involved in cell proliferation and apoptosis was up-regulated in not only biopsy samples but also in urine samples of RCC cases and is an important biomarker of malignant ccRCC [50]. miRNA let-7 has been found to be dysregulated in various types of tumors and is a generally acknowledged tumor suppressor. In a study conducted by Fedorko et al. [52], all members of miRNA let-7 were studied in the urine samples of RCC patients and controls. Higher miRNA let-7 concentrations were observed in RCC patients as compared to controls.

Investigations suggest that miRNA are potential prognostic biomarkers in RCC. These biomarkers are capable of stratifying patients and predicting the development of diseases. Of late it was reported that miR-21 and miR-221, both were overexpressed in RCC tissue samples as compared to normal samples and are associated with poor prognosis and a reduced OS of patients [53]. Overexpression of miR-221 and miR-222 is implicated with the activation of epidermal growth factor receptor (EGFR)-RAF-RAS-MEK or EFGR/MAPK pathway and its inhibition can result in reduced cell invasion capacity. Data revealed that enhanced expression of miR-221, miR-210, and miR-1233 conferred the utmost risk of renal cancer related death. Besides, 13 other miRNA (miR-9-1, miR-9-2, miR-18a, miR-21, miR-130b, miR-146b, miR-149, miR-183, miR-223, miR-335, miR-365-1, miR-365-2, and miR-625) are linked with elevated tumor re-occurrence rates [54]. Similarly, Huang et al. [55] communicated that miR-223-3p, miR-21-5p, and miR-365a-3p are associated with high re-occurrence rates and worse survival in ccRCC patients. The under expression of miR-497 was demonstrated to be associated with dreadful tumor stages and higher histological grading by Zhao et al. [56]. The major edge of miRNA as RCC biomarkers is their small size which makes them suitable for samples with low RNA quality such as body fluids or biopsy samples. Varied detection techniques like reverse transcriptase quantitative PCR (RT-qPCR), next generation sequencing (NGS) and microarray have been employed in the analysis of miRNA [57] (Table 2).

**Table 2:** miRNA in diagnosis, monitoring, and progression of RCC

**miRNA**	**Sample**	**Expression**	**Comments**		**Reference**
miR in RCC diagnosis					
miR-210/miR-210-3p	Urine	Up-regulated	Expressed in response to hypoxia mainly through HIF-1α, a key player of renal carcinogenesis miR-210 overexpression directly targets HIF-1α expression and suppresses the HIF-1α pathway activation, thereby significantly attenuating the hypoxia induced renal tubular cell apoptosis	[58, 59]	
miR-200 family (miR-200a, miR-200b, miR-200c, miR-141 and miR-429	Urine and serum	Down-regulated	Act as tumor suppressors markers Involved in the regulation of EMT, tumor metastasis, tumor stemness maintenance, and chemotherapy resistance process in cancer development	[33, 60, 61]	
miR-15a	Biopsy and urine samples	Up-regulated	Apoptosis and cell proliferation Promotes proliferation, invasion, migration, and epithelial mesenchymal transition of ccRCC cells Accelerates RCC cell viability by downregulating BTG2 and promoting the activity of the P13K/AKTsignalling pathway	[50, 62]	
miR-30c-5p	Urine exosomes	Down-regulated	Modulates the expression of HSPA5 which is correlated with the progression of ccRCC Associated with increased HIF-2α activity promoting epithelial mesenchymal transition in ccRCC	[63, 64]	
miR-497	Tissues, blood, and urine	Down-regulated	Involved in processes like inflammatory responses, malignant behavior of tumors, and epithelial-mesenchymal transformation Regulates proliferation of ccRCC via up-regulation of IL-6R	[65]	
miR-204-5p	Urinary exosomes	Down-regulated	Acts as a tumor suppressor which suppresses RCC proliferation and invasion by targeting the *RABB22A* gene	[66, 67]	
miR-200a-3p/miR-34a-5p/miR-365a-3p	Urine	Down-Regulated	-	[68]	
miR-28/miR-125/miR-27/miR-let-7f-2	Tissue	Up-regulated	Induced cell mobility and inhibited apoptosis	[69]	
miRNA in RCC monitoring					
miR-210-3p	Urine	Down-regulated in RCC follow up samples post treatment	Up-regulated miR-210 in RCC promotes cell proliferation and tumorigenesis through the epithelial mesenchymal transition pathway by targeting the *TWIST1* gene	[69, 70]	
miR-let-7d-5p/miR-152-3p/miR-30c-5p/miR-362-3p/miR-30e-3p	Urine	Down-regulated post-surgery	-	[68]	
miRNA in RCC prognosis					
miR-221	Plasma	Up-regulated	Enhances tumor cell proliferation through the angiogenesis pathway Co-related with lower OS rate in patients with metastasis Promotes cell proliferation, and mobility and inhibits cell apoptosis in 786-O and ACHN cell lines	[69, 71, 72]	
miR-122-5p/miR-206	Serum	Up-regulated	Reduced period of progression free, cancer specific, and OS in ccRCC patients	[51]	
miR-149	Plasma, serum, and urine	Down-regulated	Loss of miR-149 is linked to the gain of function of the *KCNMAI* and *LOX*	[56]	
miR-9-1, miR-9-2, miR-18a, miR-21, miR-130b, miR-146b, miR-149, miR-183, miR-223, miR-335, miR-365-1, miR-365-2 and miR-625	Plasma, serum, and urine	-	Associated with worse tumor stages and elevated tumor re-occurrence	[54]	

EMT: epithelial and mesenchymal transition; HSPA5: heat shock protein 5; *KCNMAI*: oncogenes potassium calcium-activated channelsubfamily m alpha 1; LOX: lysyl oxidase; -: blank sell

## lncRNA non-invasive RCC biomarkers

lncRNA are long RNA transcripts (approximately 200 nucleotides) without an open reading frame that are involved in biological functions like proliferation, cell differentiation, chromosome imprinting, and DNA damage response (most of which require protein interaction) [73, 74]. They regulate protein stability via RNA-protein interaction [75]. The tumor derived circulating cell free RNA molecules can be easily detected in significant amounts in body fluids and thus serve as potential diagnostic markers in tumors [76]. Over the past, there has been a surge in the data that validate the association between clinical outcomes for cancer patients and aberrant expression of lncRNA. The increase or decrease in their expression imparts to oncogenesis by affecting several cellular processes and hence they were considered notable contenders in cancer biology or RCC (Table 3). The meta-analysis study by Chen et al. [77] revealed that high expression of metastasis associated lung adenocarcinoma transcript 1 (*MALAT1*) could be considered as a biomarker for diagnosis of lymph node metastasis and distant metastasis at early stages as well as a predictor of poor survival in RCC patients. Furthermore, up-regulation of RCC related transcript-1 (*RCCRT1*), protein sprouty homolog 4 intronic transcript-1 (*SPRY4-IT1*), and *H19* have been linked with poor prognosis of RCC. It was also demonstrated that down regulation of cell adhesion molecule 1 antisense transcript-1 (*CADM1-AS1*), neuroblastoma associated transcript-1 (*NBAT-1*), and *lnc-ZNF180-2* reduced the expression of RNA in androgen independent cells. Downregulated RNA in cancer (*DRAIC*, inhibitor of cell invasion and migration) and erythrocyte membrane protein band 4.1 like 4A-antisense RNA2 (*EPB41L4A-AS2*) have been coupled with poor prognosis of RCC. Lately, the contribution of lncRNA as biomarkers of RCC was reviewed by Rysz et al. [75]. They discussed three glycolysis-related lncRNAs (*AC156455.1*, *AC009084.1*, and *LINC00342*) which allowed for the prediction of ccRCC clinical prognosis. Moreover lncRNAs, *LINC00460*, *AL139351.1*, *AC156455.1*, *AL035446.1*, *LINC02471*, *AC022509.2*, and *LINC01606* that are associated with the development and progression of ccRCCmay be implicated with DNA mismatch repair, replication of DNA and cell cycle. A cohort study of the kidney renal clear cell carcinoma (KIRC) of TCGA using Kaplan-Meier prognostic analysis and a Cox proportional hazards regression model was performed by Liu et al. [78]. They recognized 26 distinctly expressed lncRNAs (11 up-regulated and 15 down-regulated) using average linkage clustering. Further, they identified 30 statistically significant lncRNA that were strong RCC prognosis predictors. Among these 4 lncRNA specific to ccRCC (*TCL6, PVT1, MIR155HG,* and *HAR1B*), were studied to be differentially expressed and correlated with OS remarkedly [78]. Besides, recently it was reported that lncRNA *FTX* was overexpressed in RCC which enhanced the feasibility of RCC cells as well as accelerated their cell cycle progression through the miRNA sponge effect on miR-4429 [79]. They are also involved in promoting proliferative, migratory, and invasive capacities thereby upregulating ubiquitin-conjugating enzyme E2C (UBE2C). UBE2C is an essential factor for anaphase promoting complex/cyclososme (APC/C) and cell cycle regulatory E3 which is involved in the speeding up of the cell cycle through ubiquitination modification of cyclins and mitosis related factors.

**Table 3:** Diagnostic and prognostic lncRNA biomarkers of RCC

**lncRNA biomarker**	**Expression in RCC patients**	**Role in RCC**	**Reference**
*MEG3*	Down-regulation	MEG3 acts as a lncRNA tumor suppressor in various tumors through interaction with p53	[80]
*EGOT*	Down-regulation	EGOT acts as a tumor suppressor in RCC and affects RCC cell migration, invasion, and apoptosis	[81]
*MALAT1*	Up-regulation	MALAT1 overexpression enhances RCC cell proliferation, invasion and decreases cell apoptosis Increased MALAT1 expression predicted poor survival in RCC patients	[77]
*CRNDE*	Up-regulation	CRNDE-enhanced ccRCC cell migration and invasion through modulating EMT-associated genes	[33]
*LINC01510*	Down-regulation	LINC01510 when normally expressed suppresses cell proliferation by inhibiting Wnt/β-catenin signaling	[82]
*ZNF-180-2*	Up-regulation	ZNF-180-2 may regulate RNA splicing through RNA-protein interaction ZNF-180-2 allows the identification of patients with poor prognosis	[83]
*PVT1*	Up-regulation	PVT1 affects apoptosis through Mcl-1, involved in regulating cell death and comprising both pro- and antiapoptotic factor PVT1 is a good marker of worse prognosis and shorter survival of patients with higher PVT1 levels	[84]
*TCL6*	Down-regulation	The miR-155-5p targeted down-regulation of TCL6 involved activation of Src-Akt-induced EMT which is related to ccRCC progression and metastasis	[75]
*DLEU2*	Up-regulation	Stimulates tumor cell proliferation via modulating the Notch signalling pathway, or through regulation of EMT Abnormal expression of DLEU2 is associated with copy number variations and DNA methylation	[75]

MEG3: maternally expressed 3; EGOT: eosinophil granule ontogeny transcript; CRNDE: colorectal neoplasia differentially expressed; EMT: epithelial-mesenchymal transition; Mcl-1: myeloid leukemia cell differentiation protein 1; PVT1: plasmacytoma variant translocation 1; TCL6: tcellleukemia/lymphoma 6; DLEU2: deleted in lymphocytic leukemia 2

## Protein based non-invasive biomarkers for RCC

Over the past few years, several protein biomarkers have been investigated for their potential as non-invasive easily diagnosable, and early detection tools for RCC. Although there are various urinary proteins in experimentation for RCC diagnosis, e.g., carbonic anhydrase 9 (CA9), neutrophil gelatinase-associated lipocalin, Raf-kinase inhibitory protein, nuclear matrix protein-22, aquaporin-1, 14-3-3 protein β/α, perilipin-2, etc., however, none of these have been approved for clinical use due to low sensitivity and reproducibility, or due to lack of experimental validation [85]. Another study demonstrated that nicotinamide-N-methyltransferase (NNMT), secretagogin, L-plastin, neuron specific enolase (NSE), NM23, ferritin light chain, and thioredoxin peroxidase were the candidate biomarkers that were elevated in RCC tumors [79]. According to tis study, secretagogin was expressed mainly in ccRCCwhereas L-plastin and NM23 (nucleoside diphosphate kinase 1) are expressed in all types of RCC.

Besides their potential as a diagnostic tool, circulating proteins have been investigated as remarkable prognostic and predictive biomarkers for RCC. The study conducted by Peters et al. [86] demonstrated that higher CA9 serum concentrations in metastatic ccRCC patients decreased OS among patients. The expression of CA9 is regulated by the HIF1α and is known for interfering with the hypoxia process [87]. Hypoxia induced low oxygen concentration, an extracellular pH and high hydrostatic pressure helps promote angiogenesis as well as tumor growth and metastasis. As yet no reliable molecular biomarker that is able to detect the aggressiveness of RCC is available in clinical practice. However, the application of CA9 as a diagnostic biomarker for ccRCC is well ingrained with a sensitivity of 85–100%. Besides CA9, other immunomarkers such as cytokeratin 7 (CK7) and alpha-methylacyl-CoA racemase (AMACR) have been useful in the diagnosis of particularly high-grade clear cell tumors. But most procedures perform an evaluation of these immunomarkers through immunostaining of renal cancer tissues rather than in biological fluids [88].

Other proteins such as kidney injury molecule 1 (KIM1), CD27, CD70, and TNF-related apoptosis-inducing ligand (TRAIL) serve as RCC diagnostic markers and may correlate with poor survival and metastasis, thereby providing an insight into the disease progression. Further, high baseline levels of selected cytokines (IL-6, IL-8, and osteopontin) were studied to be negative prognostic factors of RCC [89]. Further, adenosine, glycosaminoglycans, tryptophan, and kynurenine are promising metabolic biomarkers for metastatic RCC [90].

## Biomarkers in targeted therapies for RCC

In the treatment of various cancers, including RCC, targeted therapy has become an encouraging approach for enhancing the survival end point. As an alternative to traditional chemotherapy which works on the mechanism of cytotoxicity and has strong side effects as well as poor selectivity, targeted therapy inhibits or prevents the growth and proliferation of tumor cells by inhibiting the correlated signal molecules [91]. Prior investigations showed that biological factors, such as VEGF and tyrosine kinase inhibitor (TKI), play vital roles in the gene targeted therapy of RCC. As discussed before, tumorigenic VEGFA is up-regulated due to the loss or silencing of the *VHL* gene in the early stages of RCC, which consequently leads to HIFα accumulation. Enhanced VEGF contributes to angiogenesis and has potential implications in clinical gene therapy for RCC [92]. Similarly, one of the three subunits of HIFα i.e. HIF2α is considered an optimum target for ccRCC. Being upstream of multiple oncogenic pathways, it is the main operator of ccRCC. Therefore, multiple VEGF and HIF2α inhibitors as well as mTOR inhibitors have been explored over the last decade as potential therapeutics for advanced and metastasised RCC. Anti-VEGF drugs include either the intravenously administered anti-VEGF antibodies such as bevacizumab combined with interferon alfa-2a or the orally administrable TKI that target the circulating VEGF or VEGF receptors (VEGFRs) such as axitinib, cabozantinib, lenvatinib, pazopanib, sorafenib, sunitinib, and tivozanib, while mTOR inhibitors include temsirolimus and everolimus [93]. These TKI have demonstrated notable activity against RCC in randomized clinical trials. Sunitinib and pazopanib were the first to be approved for the frontline treatment of metastasised RCC [94]. The TKI sunitinib and sorafenib target and inhibit the VEGF receptor, platelet derived growth factor receptor (PDGFR), c-Kit as well as fms-related receptor tyrosine kinase (Flt3) and hence act as antiangiogenic therapeutics [95]. Bevacizumab which was approved in 2004, is the VEGF blocking antibody that has validated the principle of anti-angiogenesis for tumor therapy clinically [96]

Although, TKI have been studied as a cornerstone treatment of RCC with sunitinib being the preferred first line treatment for all cases. However, in recent times TKI monotherapy is not recommended for metastasized RCC as a preferred treatment. Instead, TKIs have been efficiently applied to treat patients in combination with immunotherapies [97]. A study by Hirsch et al. [98] reported that using VEGFR-TKI (vascular endothelial growth factor receptor tyrosine kinase inhibitor) along with immune checkpoint inhibitors (ICIs) has become a new standard of care in patients with advanced RCC. As a result of clinical investigations, a combination of pembrolizumab plus axitinib and avelumab plus axitinib has been approved as the preferred treatment of patients with advanced RCC. These investigations tested different associations of anti-angiogenic therapies such as VEGFR-TKI or bevacizumab jointly with ICIs like programmed cell death protein 1 (PD-1) or programmed cell death ligand 1 (PD-L1) inhibitors. The anti-angiogenic therapies hinder the immunosuppressive effect created by VEGF or its receptors by enhanced infiltration of mature dendritic cells and effector T cells into the tumor cells and reduced infiltration of regulatory T cells and myeloid derived suppressor cells. This immunomodulatory effect of anti-angiogenic therapies in combination with ICI hence provides enhanced activity against RCC (Table 4).

**Table 4:** TKI based therapies of RCC and their clinical trials

**Drug**	**Trial**	**Findings of the trial**	**Reference**
Pazopanib	Double blind, randomized, placebo-controlled phase III trial (sample size: 435)	Treatment with pazopanib significantly prolonged median PFS in comparison to placebo (9.2 months *vs.* 4.2 months; HR = 0.46, *P* < 0.001) in patients with locally advanced or metastasised RCC	[97]
Cabozantinib	The Alliance A031203 CABOSUN trial	Monotherapy with 60 mg of daily cabozantinib compared to sunitinib standard therapy (50 mg once per day; 4 weeks on, 2 weeks off) resulted in an increased ORR (33% *vs.* 12%) and a remarkable PFS benefit (8.2 months *vs.* 5.6 months)	[99]
Sorafenib	TARGET trial (sample size: 903)	Sorafenib displayed superiority as indicated by the median PFS (5.5 months *vs.* 2.8 months) in the placebo group with an HR of 0.44 (*P* < 0.01)	[100, 101]
	Tivo-1 trial	Compared to tivozanib, sorafenib therapy displayed worse PFS but similar OS	
Axitinib	Phase-3 AXIS trial (sample size: 723)	In comparison to sorafenib, median PFS was significantly longer in metastasised RCC patients treated with axitinib (8.3 months *vs.* 5.7 months, HR = 0.66, *P* < 0.0001) As evident with fewer AE-related treatment discontinuation, axitinib was unique due to less severe side effects	[102]
	Phase II AXIPAP trial	The overall median PFS, median PFS for type 1 pRCC, and median PFS for type 2 pRCC were 6.6 months, 6.7 months, and 6.2 months, respectively The median overall OS was 18.9 months Type 2 pRCC showed a rather high 36% ORR	[103]
Cabozantinib *vs.* Sunitinib	SWOG PAPMET trial	Cabozantinib displayed a PFS benefit (9.0 months *vs.* 5.6 months; HR = 0.60) and higher ORR (23% *vs.* 4%) over sunitinib	[104]
Pembrolizumab + lenvatinib *vs.* sunitinib	CLEAR trial	Pembrolizumab + lenvatinb demonstrated a longer median PFS (23.9 months *vs.* 9.2 months; HR = 0.39) over sunitinib Pembrolizumab + lenvatinib-treated patients had improved OS and higher ORR (71.0% *vs.* 36.1%) over sunitinib-treated patients The risk of death observed was 34% lower in patients treated with pembrolizumab + lenvatinib	[101]
Lenvatinib/everolimus *vs.* lenvatinib + everolimus	Phase-II trial	A longer PFS was observed for longer PFS for lenvatinib and everolimus in combination and single agent lenvatinib when compared to everolimus, respectively The longest median PFS of 14.6 months was obtained with combinational therapy of lenvatinib and everolimus Lenvatinib monotherapy displayed a PFS of 7.4 months and a hazard ratio of 0.66 Severe AE was observed in 71% and 79% of those receiving lenvatinib combination- and single-agent-therapy, respectively	[105]
Atezolizumab + bevacizumab *vs.* sunitinib	Phase III IMmotion151 trial	Patients receiving atezolizumab + bevacizumab, reported greater symptom improvements *vs.* sunitinib with an objective response of 49% *vs.* 14%, including complete responses of 10% *vs.* 3%	[102]
Cabozantinib and nivolumab *vs.* sunitinib	CheckMate 9ER trial	A better OS rate, PFS, and a more likely response than sunitinib monotherapy was demonstrated with a combination of cabozantinib and nivolumab	[106]

PFS: progression free survival; HR: hazard ratio; ORR: objective response rate; AE: adverse events

Apart from VEGFA, another gene considered as a potential portending gene for RCC diagnosis and targeted therapy is *DLL4*. Analysis of several malignant tissues reveal the enhanced expression of DLL4 in RCC due to increasing invasion grade and is found to be associated with tumor size, clinical stage, and lymph node metastasis. DLL4/Notch signalling is a major pathway that is critically involved in normal vascular development and pathological angiogenesis [13]. DLL4 is expressed in the vascular endothelium of ccRCC which on one hand activates VEGF thereby promoting angiogenesis and on the other hand activate Notch signalling in tumor cells thus inducing hematogenous metastasis [107]. On the basis of their characteristics, a bunch of DLL-targeted therapies have been proposed. Amongst these anti-DLL4 humanized antibodies or bispecific monoclonal antibodies targeting both human DLL4 and human VEGF such as navicixizumab (knob-in-hole), HD-105 (scFv_2_-Fc), HB-32 (CrossMAb) and ABT-165 (DVD-Ig) have been established and are under clinical trials to assess for their safety and efficacy. Besides, recombinant proteins and miRNA have also been studied to modulate the activity of DLL4 [108]. Di Martino et al. [53], recently discussed that utilizing miR-221 inhibitor was able to increase molecular tumor suppressor tissue inhibitor of metalloproteinase 2 (TIMP2) levels thus improving the cell membrane integrity and hence contributes to the inhibition of kidney cancer. The evidences obtained from numerous investigations engendered the use of miR-221 as the chief therapeutic target in treating RCC.

Recently it was revealed that inhibitors of the NOTCH LY-3039478 results in an increase in survival in ccRCC xenografts, indicating an alternative treatment for RCC [109]. It was reported that several DNA methylation inhibiting drugs such as azacytidine oligonucleotide MG98 as well as drugs belonging to histone deacetylase inhibitors (HDACis) class such as vorinostat, panobinostat, romidepsin, and belinostat are in phase I/II clinical trials and are being considered for RCC targeted therapy [109].

Even though a myriad of novel targeted therapies for RCC have become evident over the past few years, however a fine percentage of treated patients exhibit progressive disease due to acquired resistances to these therapies. Instead, these therapies may expose patients to unnecessary toxic effects along with burdening society with the financial impact. Hence a major challenge in this regard remains the appropriate selection of targeted therapies for any individual patient with this disease. Biomarkers for RCC can act as predictive factors that can predict the response to a specific treatment in any given patient. Besides, mi-RNA and DNA methylation biomarkers, certain circulating cytokines and angiogenic factors (CAFs) have been studied to predict the response of VEGFR and mTOR inhibitor targeted therapies. The following table summarizes the various non-invasive predictive biomarkers for RCC.

Lately, a very interesting biomarker that involved the gut microbiome was found by Xu et al. [110]. It was demonstrated that certain bacteria in the gut enhance the chances of response to immunotherapy. The lately published phase 1 trials [NCT03829111] reported that the addition of CBN588, a gut microbiome product to patients who received ipilimumab plus nivolumab enhanced the response rate to the therapy and also improved progression-free survival in patients. Hence this indicated the role of the gut microbiome in predicting response to RCC targeted therapies. A phase III study CheckMate-025 identified certain circulating metabolic pathway substrates in RCC patients. They demonstrated that a decrease in the kynurenine/tryptophan ratio over time during treatment with ICI was associated with improved OS. Also, a low level of adenosine in patients treated with nivolumab was associated with a better response when treated with the checkpoint inhibitor [1] (Table 5).

**Table 5:** Predictive non-invasive biomarkers for RCC

**Biomarker**	**Associated outcomes**	**Reference**
LAD1/CST6/NEFH	DNA hypermethylation of NEFH, LAD1, and CST6 CpG is significantly associated with poor response to antiangiogenic therapies in advanced RCC	[38]
FOXP3	The methylation of FOXP3 is a marker of regulatory T cells. The regulatory T cell population was significantly expanded in non-responders to immunotherapy as compared to therapy-responding patients	[111]
miR-183	miR-183 predicts the response of renal cancer cells to NK cell therapy Primary renal cancer cells with under-expressed miR-183 were more responsive to NK cell therapy	[112]
miR-484/miR-155-5p	Patients with significantly up-regulated levels of miR-484/miR-155-5p are refractory to sunitinib treatment	[113]
miR-942	miR-942 was observed to be overexpressed in sunitinib resistant cell line Caki-2 and hence is a predictor of sunitinib efficacy	[114]
miR-22; miR-24; miR-99a; miR-194; miR-214; miR-335; miR-339; miR-708	These miRNA were specifically induced in long responders to nivolumab but were silenced to baseline in patients with metastatic ccRCC	[49]
miR-133a; miR-628-5p	The sunitinib resistant cells expressed greater levels of miR-133a and miR-628-5p compared to sunitinib sensitive cells	[115]
GATA1/miR-885-5P/PLIN3 axis	Sunitinib resistant cell lines displayed significantly lower levels of miR-885-5p. Reduced expression of GATA1 down regulates the expression of miR-885-5p which enhances the expression of PLIN3 and induces resistance to sunitinib	[115]
VEGF-A, SDF-1, sVEGFR1, sVEGFR2, sVEGFR3	Baseline high serum levels of VEGF-A, SDF-1, and sVEGFR1 as well as low levels of sVEGFR2 are associated with a shorter PFS and OS during sunitinib treatment Low baseline plasma levels of sVEGFR3 were markedly linked to improved response to sunitinib Decreased plasma levels of VEGF-A were observed in responders to atezolizumab monotherapy	[89]
IL-6	Up regulation of plasma IL-6 levels represents an important resistance to sunitinib Low baseline plasma IL-6 levels are associated with significant response to sunitinib and improved PFS	[89]
LDH	Elevated baseline serum LDH in patients is associated with Increased OS in temsirolimus *vs.* IFN-α recipients	[116]

NK: natural killer; CST6: cystatin 6; FOXP3: forkhead box transcriptional factor; PLIN3: perilipin 3; SDF-1: stromal cell derived factor-1; sVEGFRs1: soluble VEGFR 1; LDH: lactate dehydrogenase

## Conclusions

Over the past two decades, there has been an ideal transition in the management of renal carcinomas with the approval of new diagnostic tools and therapies. As we are advancing to the era of ‘precision medicine’ the understanding of prospective biomarkers for diagnosis and therapy response and their endowment to tumorigenesis are becoming highly applicable in cancer management. The development of non-invasive epigenetic biomarkers such as DNA methylation markers, miRNA, lncRNA, or protein biomarkers have become useful for early cancer diagnosis, prediction of cancer prognosis, and response to targeted therapies as well as determination of OS in RCC patients. However, to date hardly any liquid biomarkers have been approved in RCC regardless of the demand to diagnose, predict and monitor response non-invasively to tailor treatment choices. Sample acquisition, storage, and analysis are the major limitations in the routine use of such biomarkers. Further, the identification and validation of RCC biomarkers is at a preliminary stage. Consequently, variability in the pre-analytical and the analytical phase could influence the reproducibility and precision of biomarkers thereby limiting their development and application. Future investigation of these recently identified molecular events that initiate and maintain molecular alterations and epigenetic gene silencing will assist in clarifying the relevance of different molecular signalling pathways and in the development of clinical cancer prevention and treatment strategies. Moreover, in order to achieve reliable and accurate quantification of these biomarkers, strict standardization of assay procedures should be endorsed as well as larger validation studies are needed in clinical trials controlling all variables. Identification of novel biomarkers will further open a new era of tailored medicine for RCC.

## Abbreviations

AKT: v-akt murine thymoma viral oncogene homolog
APC: adenomatous polyposis coli
CA9: carbonic anhydrase 9
ccRCC: clear cell renal cell carcinoma
CDH1: cadherin 1
CDKN2A: cyclin dependent kinase inhibitor 2A
chRCC: chromophobe renal cell carcinoma
DLL4: delta like canonical notch ligand 4
EGFR: epidermal growth factor receptor
EMT: epithelial-mesenchymal transition
ERK2: extracellular signal-regulated kinase 2
GATA5: GATA binding protein 5
GREM1: gremilin 1
HIF: hypoxia inducing factor
ICIs: immune checkpoint inhibitors
LAD1: laudinin 1
lncRNA: long noncoding RNA
MALAT1: metastasis associated lung adenocarcinoma transcript 1
MET: mesenchymal-epithelial transition factor
MGMT: O^6^-methyl-guanine-DNA-methyltransferase
miRNA: microRNA
MSP: methylation specific polymerase chain reaction
mTOR: mammalian target of rapamycin
NEFH: neurofilament heavy polypeptide
NRF2: nuclear factor erythroid 2-related factor 2
NRTs: non-cancerous renal tissues
ORR: objective response rate
OS: overall survival
PCDH17: rotocadherin 17
PCDH8: protocadherin 8
PCR: polymerase chain reaction
PFS: progression free survival
pRCC: papillary renal cell carcinoma
PVT1: plasmacytoma variant translocation 1
qPCR: quantitative polymerase chain reaction
RASSF1A: Ras association domain family 1 isoform A
RCC: renal cell carcinoma
TIMP3: tissue inhibitor of metalloproteinase-3
TJP2: tight junction protein 2
TKI: tyrosine kinase inhibitor
VEGF: vascular endothelial growth factor
VEGFRs: vascular endothelial growth factor receptors
VHL: von Hoppel Lindau
ZNF677: Zinc finger protein 677

## Declarations

### Copyright

© The Author(s) 2023.
